# Increased susceptibility of transgenic mice expressing human PrP to experimental sheep bovine spongiform encephalopathy is not due to increased agent titre in sheep brain tissue

**DOI:** 10.1099/vir.0.065730-0

**Published:** 2014-08

**Authors:** Chris Plinston, Patricia Hart, Nora Hunter, Jean C. Manson, Rona M. Barron

**Affiliations:** The Roslin Institute & R(D)SVS, University of Edinburgh, Easter Bush, Midlothian EH25 9RG, UK

## Abstract

Bovine spongiform encephalopathy (BSE) in cattle and variant Creutzfeldt–Jakob disease in humans have previously been shown to be caused by the same strain of transmissible spongiform encephalopathy agent. It is hypothesized that the agent spread to humans following consumption of food products prepared from infected cattle. Despite evidence supporting zoonotic transmission, mouse models expressing human prion protein (HuTg) have consistently shown poor transmission rates when inoculated with cattle BSE. Higher rates of transmission have however been observed when these mice are exposed to BSE that has been experimentally transmitted through sheep or goats, indicating that humans may potentially be more susceptible to BSE from small ruminants. Here we demonstrate that increased transmissibility of small ruminant BSE to HuTg mice was not due to replication of higher levels of infectivity in sheep brain tissue, and is instead due to other specific changes in the infectious agent.

Transmissible spongiform encephalopathy (TSE) diseases (also known as prion diseases) are fatal, infectious neurodegenerative diseases of animals, and include bovine spongiform encephalopathy (BSE) in cattle, scrapie in sheep and Creutzfeldt–Jakob disease (CJD) in humans. TSEs are unusual diseases, as the infectious agent is thought to be composed solely of a misfolded conformer of the normal host glycoprotein PrP^C^ (Prusiner, 1982). The abnormally folded conformer (PrP^Sc^) is present in infected tissues of the central nervous system (CNS) and lymphoreticular system, and propagates via a templated seeding mechanism, where the abnormal protein binds to and converts normal PrP^C^ into the abnormal conformation (Caughey, 2001).

Ruminant TSEs were for many years thought to be of low risk to humans due to the lack of association between incidence of natural scrapie in sheep and CJD in humans. However, in 1996, a new variant of CJD (vCJD) was identified in humans that presented with a different clinical and pathological phenotype (Will *et al.*, 1996). In particular, the affected individuals were of a considerably younger age than normally expected for CJD. Strain typing studies performed in mouse panels revealed that the strain of agent responsible for vCJD was the same as that responsible for the recent epidemic of BSE in UK cattle (Bruce *et al.*, 1997; Hill *et al.*, 1997). It was therefore concluded that a zoonotic transmission of the BSE agent had occurred from cows to humans via food products prepared from infected cattle. To date, there have been 177 deaths due to vCJD in the UK since it was first identified in 1995 (data provided by the National CJD Surveillance and Research Unit, Western General Hospital, Edinburgh), and all clinical cases have occurred in individuals who are homozygous for methionine at codon 129 in the human PrP gene (Zeidler *et al.*, 1997).

As TSEs present with long preclinical incubation times, establishing routes and possible frequency of exposure in humans has been difficult, and all studies have been performed as retrospective analyses on post-mortem tissue. In order to model disease transmission in a controllable laboratory situation, transgenic mouse models that express the human PrP gene instead of the mouse PrP gene have been engineered. These models allowed experimental challenge with cattle BSE (caBSE) in the presence of human PrP, and it was hoped these models would provide evidence on routes and risks of exposure of humans to caBSE. Considerable variation in susceptibility was observed between different transgenic lines with various constructs and protein expression levels. However, in general, transmission rates in mice overexpressing human 129M-PrP were found to be low (0–30 %) (Asante *et al.*, 2002; Béringue *et al.*, 2008; Padilla *et al.*, 2011), and caBSE failed to cause disease in knock-in human PrP transgenic mice (HuMM) that expressed human PrP at the same levels at WT mouse PrP (Bishop *et al.*, 2006). Together these experiments indicated that the transmission barrier between humans and cattle was high and that the disease was not easily transmitted to humans. This may reflect the low number of clinical cases of vCJD (*n* = 177) compared with the size of the UK population that may have been exposed to the agent in the 1980s and 1990s, and the predicted number of possible silent carriers in the general population, evaluated from analysis of anonymized lymphoid tissue (de Marco *et al.*, 2010; Gill *et al.*, 2013; Hilton *et al.*, 2002).

Although sheep and goats were exposed to the same sources of contaminated feed (but in lower quantities) as cattle during the BSE epidemic (Ferguson *et al.*, 2002), no natural cases of BSE have been documented in sheep. Two cases of natural BSE infection have been identified in goats (Eloit *et al.*, 2005; Jeffrey *et al.*, 2006; Spiropoulos *et al.*, 2011), and sheep and goats have been shown to be susceptible to infection via intracerebral, oral and intravenous experimental exposure (Foster *et al.*, 1993, 1996; Houston *et al.*, 2000). Hence, BSE infection of small ruminants is possible. In previous studies, we and others have shown that experimental sheep BSE (exp-shBSE) and experimental goat BSE (exp-gtBSE) are more easily transmissible than caBSE to transgenic mice expressing human PrP (Padilla *et al.*, 2011; Plinston *et al.*, 2011; Wilson *et al.*, 2013). In two separate challenges from individual infected animals, we have shown TSE disease pathology in 40–70 % of recipient knock-in human PrP transgenic mice homozygous for methionine at codon 129 (HuMM mice), whereas the same animals showed no signs of disease when inoculated with caBSE (Bishop *et al.*, 2006; Plinston *et al.*, 2011; Wilson *et al.*, 2013). In mice overexpressing human 129M-PrP, both sheep and goat BSE have been shown to transmit with 100 % efficiency, compared with 0–30 % efficiency with cattle BSE (Padilla *et al.*, 2011).

These observations are perplexing, as sheep and goat scrapie do not transmit to HuMM transgenic mice (Plinston *et al.*, 2011; Wilson *et al.*, 2012, 2013), indicating the increased susceptibility is not due simply to ovine/caprine PrP compatibility with human PrP. Another possible explanation is that sheep and goats are able to replicate the BSE agent to a higher titre in the CNS, and this results in a higher dose of agent being received by the HuMM transgenic mice. In order to examine this possibility, we performed titration bioassay of the caBSE brainstem pool previously used to challenge HuMM mice (Bishop *et al.*, 2006), and also brainstem from a case of exp-shBSE. Due to the apparent absence of a species barrier to cattle and sheep BSE on transmission to bovine transgenic mice (Espinosa *et al.*, 2007), titrations were performed in knock-in bovine PrP transgenic mice (Bov6) (Bishop *et al.*, 2006) to maximize transmission efficiency and allow direct comparison of titre. Bov6 Tg mice were maintained on the same genetic background as the HuMM mice and 129/Ola WT controls, also allowing direct comparison of the effect of bovine, human or murine PrP on transmissibility of BSE. Brain homogenate (10 %) was prepared from a caBSE brainstem pool (BBP 12/92 supplied by the Animal Health and Veterinary Laboratories Agency) (Bishop *et al.*, 2006; Plinston *et al.*, 2011), and from exp-shBSE brainstem tissue (Roslin; AHQ/AHQ J2771 sheep infected orally with BSE cattle brain; incubation time 559 days). Previous work by us and by others (Buschmann & Groschup, 2005; Safar *et al.*, 2002) has shown no difference in titre of BSE pools versus individual brains. The brainstem pool was thus selected for titration as this material had previously been used to challenge Bov6 and HuTg mice (Bishop *et al.*, 2006). Each inoculum was used to prepare serial dilutions from 10^−2^ to 10^−7^ in sterile saline. Each dilution was inoculated (20 µl, intracerebrally) into groups of 12 Bov6 transgenic mice. The 10^−1^ homogenate was also inoculated into control 129/Ola mice and HuMM transgenic mice, to ensure the exp-shBSE inoculum used was able to cause the same TSE pathology in HuMM transgenic mice as observed previously. All animals were monitored daily and scored weekly for signs of clinical disease and culled either due to intercurrent illness or at a pre-defined clinical end point. Tissue from each mouse was analysed post-mortem for presence of TSE-associated vacuolation (Fraser & Dickinson, 1967) and PrP deposition using MAb6H4. All experiments were performed under licence from the UK Home Office and in accordance with the UK Animals (Scientific Procedures) Act, 1986.

For the purposes of titre determination, we classed all animals showing both clinical signs of TSE and TSE-associated pathology as positive transmission. Survivors were classed as animals that outlived the last positive case in each dilution group. Animals that were culled early due to intercurrent illness before the last TSE-positive case in each group were not included in the analysis. Although clinical signs of disease in all Bov6 mice were similar, regardless of the source of BSE inoculum, incubation times for exp-shBSE in each dilution group were consistently shorter than the corresponding dilution of caBSE (Table 1). However infectious titres calculated using the Spearman–Karber method (Hamilton *et al.*, 1977) were similar for both inocula; 10^5.1^ ID_50_ g^−1^ tissue (median infectious dose) for caBSE (95 % confidence interval, 10^4.8^–10^5.3^) and 10^5.0^ ID_50_ g^−1^ tissue for exp-shBSE (95 % confidence interval, 10^4.7^–10^5.4^). The difference of less than half a log between titres was not statistically significant (*P* = 0.99985; *t*-test). Several survivors were found to show TSE pathology (vacuolation and/or PrP deposition) when culled due to intercurrent illness after the last clinical positive animal in each group. Titre calculations based on TSE pathology alone also showed no significant difference between caBSE and exp-shBSE titres (*P* = 0.966; *t*-test). While no signs of disease were observed in HuMM mice inoculated with caBSE (Bishop *et al.*, 2006), 7/12 HuMM mice that received the exp-shBSE inoculum showed TSE related pathology (PrP deposition and/or vacuolation), confirming previous reports (Padilla *et al.*, 2011; Plinston *et al.*, 2011).

**Table 1.** Infectivity titres in cattle and sheep BSE brainstem

		Cattle BSE brainstem pool		Experimental sheep BSE brainstem	
**Recipient**	**Dilution**	TSE positive*	**Incubation time (days±sem)†**	TSE positive*	**Incubation time (days±sem)†**
HuMM	10^−1^	0/18‡	na	0/12	na
129/Ola	10^−1^	11/11	405±26	9/11	378±25
Bov6	10^−1^	10/10	598±20	11/11	486±9
Bov6	10^−2^	4/4	621±10	7/8	501±22
Bov6	10^−3^	7/8	657±20	7/8	527±13
Bov6	10^−4^	0/12	na	1/11	483
Bov6	10^−5^	0/12	na	0/12	na
Bov6	10^−6^	0/12	na	0/12	na
Bov6	10^−7^	0/12	na	0/12	na

na, No data.

* Animals showing both clinical signs of TSE and TSE-associated pathology.

† Incubation time calculated from animals showing both clinical signs of TSE and TSE pathology.

‡ Data from Bishop *et al.* (2006) shown for comparison.

Infectivity levels in brain tissue from sheep infected with BSE have been previously analysed by comparative titration in ARQ/ARQ Romney sheep and RIII mice (González *et al.*, 2009). Titres reported in RIII mice (10^5^ ID_50_ g^−1^) and Romney sheep (10^5.4^ ID_50_ g^−1^) in the study by Gonzalez *et al.* (2009) were equivalent to those measured here in Bov6 mice (10^5.0^ ID_50_ g^−1^). Incubation times for 10^−1^ dilution in RIII mice (389 days) (González *et al.*, 2009) and 129/Ola mice (378 +/−25 days; Table 1) were similar, indicating comparable levels of infectivity and consistent titres as measured in sheep, WT mice and bovine PrP transgenic mice. The reasons for the increased transmissibility of BSE to HuMM mice following passage in sheep and goats are still unclear. Previous studies have shown an apparent increased virulence of exp-shBSE compared with caBSE following transmission to transgenic mice overexpressing bovine or porcine PrP (Espinosa *et al.*, 2009, 2007). More recently, it has been shown that exp-shBSE can amplify all ovine and bovine PrP^C^ allelic variants by protein misfolding cyclic amplification (PMCA) more efficiently than sheep scrapie (Priem *et al.*, 2014). Importantly, these and other studies have also shown no change in BSE agent characteristics or PrP^Sc^ glycoform following passage through sheep (Espinosa *et al.*, 2007; González *et al.*, 2009; Padilla *et al.*, 2011; Plinston *et al.*, 2011). Levels of proteinase K-resistant PrP (PrP-res)were found to be lower in exp-shBSE tissue than caBSE tissue (Padilla *et al.*, 2011; Plinston *et al.*, 2011), yet data here have established no significant difference in the level of infectivity present in the exp-shBSE and caBSE infected brainstem. It is possible that the ovine/caprine PrP sequence is required to allow a more efficient interaction of the BSE agent with human 129M-PrP, however it is important to note that no disease transmission has been observed in HuMM mice following inoculation with several isolates of natural and atypical sheep scrapie and goat scrapie (Wilson *et al.*, 2013, 2012), indicating that it is not simply the presence of the ovine or caprine PrP sequence that facilitates transmission to animals expressing human PrP. In addition, *in vitro* amplification studies have shown that human 129M-PrP^C^ is converted to PrP-res with equal efficiency by BSE PrP^Sc^ from both cattle and sheep brain tissue (Jones *et al.*, 2009), indicating that human 129M-PrP can interact and convert equally well with both sheep and cattle BSE PrP-res in a cell-free environment.

Together, these data show that the increased transmissibility of exp-shBSE and exp-gtBSE to mice expressing human 129M PrP is not due solely to PrP sequence, infectivity level or the convertibility of human 129M-PrP by ovine/caprine prions. The increased transmissibility of exp-shBSE in HuTg mice must therefore be due to other specific changes in the agent and its ability to interact with the host. These may include differences in cellular factors such as routing of the inoculum and trafficking of PrP-res, or the composition and interaction of specific conformers of abnormal PrP in the inoculum. The identification of such factors that are critical for zoonosis may aid in the assessment of future TSE agent outbreaks and the associated risk to humans from these isolates. As long as TSE agents remain in the environment, the opportunity for cross-species transmission to occur remains. Our data have shown that such cross-species transmission events can have major effects on the host range and transmissibility of different TSE agents. In particular we have established that the emergence or re-emergence of BSE in small ruminants could have serious public health implications. There is therefore a strong requirement to continue surveillance for new emerging TSE strains, or for the appearance of old strains in new hosts, and quickly assess the risks of zoonosis.
